# Description of a New Species and Revised Key to the *Hydrometra* Latreille, 1797 (Hemiptera: Gerromorpha: Hydrometridae) Recorded from Brazil †

**DOI:** 10.3390/ani15172468

**Published:** 2025-08-22

**Authors:** Carla Fernanda Burguez Floriano, Isabelle da Rocha Silva Cordeiro, Juliana Mourão dos Santos Rodrigues, Nathália de Oliveira Paiva, Ana Carolina Passos, Felipe Ferraz Figueiredo Moreira

**Affiliations:** Laboratório de Entomologia, Instituto Oswaldo Cruz, Fundação Oswaldo Cruz, Rio de Janeiro 21040-360, Brazil; rocha.belle@gmail.com (I.d.R.S.C.); julianamourao@yahoo.com.br (J.M.d.S.R.); nathaliapaiva.93@gmail.com (N.d.O.P.); anacpca@gmail.com (A.C.P.); ppmeiameiameia@gmail.com (F.F.F.M.)

**Keywords:** aquatic insects, biodiversity, identification, Neotropical Region, semiaquatic bugs, taxonomy, water measurers

## Abstract

Hydrometridae (Hemiptera: Heteroptera: Gerromorpha) is represented in Brazil by 17 species, belonging to six genera and three subfamilies: *Veliometra* Andersen, 1977 (Heterocleptinae); *Bacillometra* Esaki, 1927, *Cephalometra* Polhemus & Ferreira, 2018, *Hydrometra* Latreille, 1797, *Spelaeometra* Polhemus & Ferreira, 2018 (Hydrometrinae); and *Limnobatodes* Hussey, 1925 (Limnobatodinae). *Hydrometra* is the richest genus in the country, with 11 recorded species. Here, we describe *H. perobas* Cordeiro, Floriano & Moreira, sp. nov., collected in the buffer zone of Reserva Biológica das Perobas, a protected area within the Atlantic Forest biome, state of Paraná, southern Brazil. We also provide a revised key to the species of *Hydrometra* recorded from Brazil.

## Simple Summary

Aquatic insects are crucial in freshwater ecosystems, serving as food and as nutrient recyclers. Water measurers (Insecta: Hemiptera: Hydrometridae: *Hydrometra*) inhabit the edges of water bodies, especially in tropical regions. This study describes a new *Hydrometra* from Paraná, increasing the number of species in Brazil from 11 to 12.

## 1. Introduction

Gerromorpha (Hemiptera: Heteroptera) comprises more than 2100 species distributed throughout all continents, except Antarctica. They are currently divided into the following eight families: Gerridae, Hebridae, Hermatobatidae, Hydrometridae, Macroveliidae, Mesoveliidae, Paraphrynoveliidae and Veliidae [1,2,3]. Hydrometridae includes over 125 known species, of which 52 have been recorded from the Neotropical Region. The latter belong to the following seven genera and three subfamilies: *Veliometra* Andersen, 1977 (Heterocleptinae); *Bacillometra* Esaki, 1927, *Bacillometroides* Polhemus & Polhemus, 2010, *Cephalometra* Polhemus & Ferreira, 2018, *Hydrometra* Latreille, 1797, *Spelaeometra* Polhemus & Ferreira, 2018 (Hydrometrinae); and *Limnobatodes* Hussey, 1925 (Limnobatodinae) [4,5,6,7]. It has been recently found [3] that *Veliometra* is closer to *Hebrus* Curtis, 1833 (Hebridae) than to *Hydrometra*, but no classification changes have been proposed due to the lack of other Hydrometridae genera in the analysis.

*Hydrometra* can be distinguished from other genera of the family by the body longer than 6.00 mm; the very long anteocular region of the head; the eyes placed closer to the posterior than to the anterior margin of the head; the apical pretarsal claws; and the meso- and metasterna without longitudinal grooves. They can be found along the margins of rivers, streams, lakes, ponds and pools, as well as in sheltered places on moist soil near water bodies [1,4,6].

In the last decade, five new Neotropical species of *Hydrometra* were described. Three of them have been collected in Colombia (*H. colombiana* Martínez & Galindo-Malagón, 2019, *H. quimbaya* Martínez & Galindo-Malagón, 2019, and *H. tayrona* Martínez & Galindo-Malagón, 2019), whereas two have been discovered in Brazil (*H. ruschii* Cordeiro, Rodrigues & Moreira 2023 and *H. tuberculata* Cordeiro, Rodrigues & Moreira 2023) [6,7].

Here, we describe a new species of *Hydrometra* from the buffer zone of Reserva Biológica das Perobas, state of Paraná, southern Brazil, and provide a revised key to the species recorded from the country.

## 2. Materials and Methods

Material examined is deposited in the Coleção Entomológica do Instituto Oswaldo Cruz, Fundação Oswaldo Cruz, Rio de Janeiro, Brazil (CEIOC). We used dry-pinned specimens for descriptions and photographs. For the photographs, we used a Leica M205 C stereomicroscope coupled with a Leica DMC 2900 digital camera (Leica Imaging Systems Ltd., Wetzlar, Germany), and the Leica Application Suite V4.7. We edited the figures with Adobe Photoshop CS5. We produced maps using QGIS 3.2.2.

We followed a previous taxonomic work [7] in our description and used the following abbreviations for measurements: body length (BL), head length (HL), anteocular length (ANTL), postocular length (POSTL), maximum anteocular width (ANTWmax), minimum anteocular width (ANTWmin), maximum postocular width (POSTWmax), minimum postocular width (POSTWmin), clypeus length (CLL), maximum clypeus width (CLWmax), head width through eyes (HW), interocular width (IOW), ocular length (OL), maximum eye width (EYE), length of antennomeres I–IV (ANT: I, II, III, IV), length of labial articles (LBL: I–II, III, IV), pronotum length at midline (PL), maximum pronotum width (PW), wing length (WiL), wing width (WiW), forecoxa/midcoxa distance (DIST1), midcoxa/hindcoxa distance (DIST2), femoral length (FEM), tibial length (TIB), length of tarsomeres I–III (TAR: I, II, III), abdominal length (ABL), maximum abdominal width (ABWmax), minimum abdominal width (ABWmin), length of abdominal mediotergites (TERL: I, II, III, IV, V, VI, VII), width of abdominal mediotergites (TERW: I, II, III, IV, V, VI, VII), length of abdominal segment VIII (SVIII), and length of posterior process of abdominal segment VIII (PP). All measurements are given in millimeters.

## 3. Results

**Figure 1:**
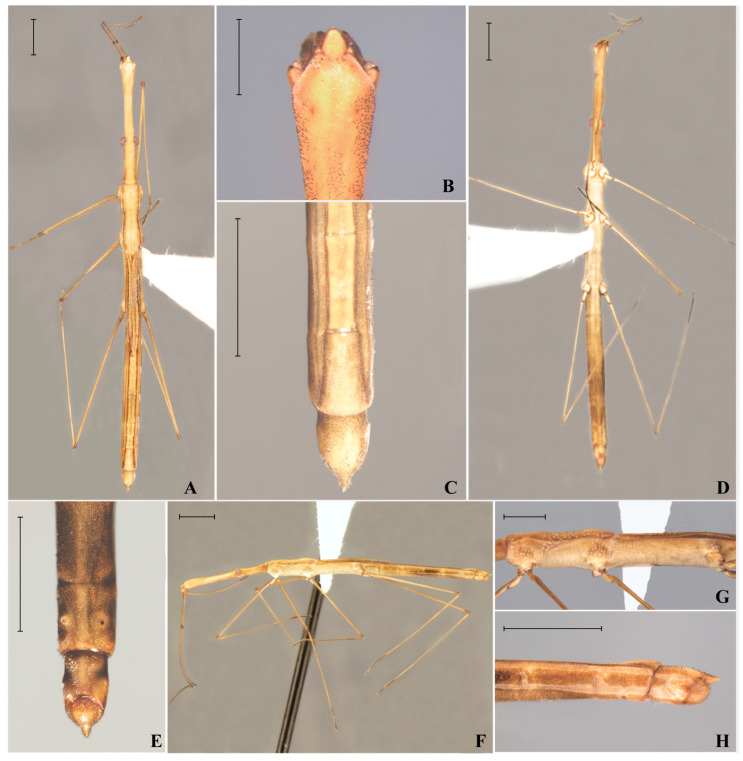
*Hydrometra perobas* Cordeiro, Floriano & Moreira, sp. nov., micropterous male, holotype. (**A**) Habitus, dorsal view. (**B**) Anterior portion of head, dorsal view. (**C**) Apex of abdomen, dorsal view. (**D**) Habitus, ventral view. (**E**) Apex of abdomen, ventral view. (**F**) Habitus, lateral view. (**G**) Thorax, lateral view. (**H**) Apex of abdomen, lateral view. Scale bars—sub-figures (**A**,**D**,**F**): 2.00 mm; sub-figure (**B**): 0.50 mm; sub-figures (**C**,**E**,**F**,**G**): 1.00 mm.

*Hydrometra perobas* Cordeiro, Floriano & Moreira, sp. nov. (Figure 1).

urn:lsid:zoobank.org:act:9FA29A0F-36F9-42FA-953C-0891F4C73160

**Figure 2:**
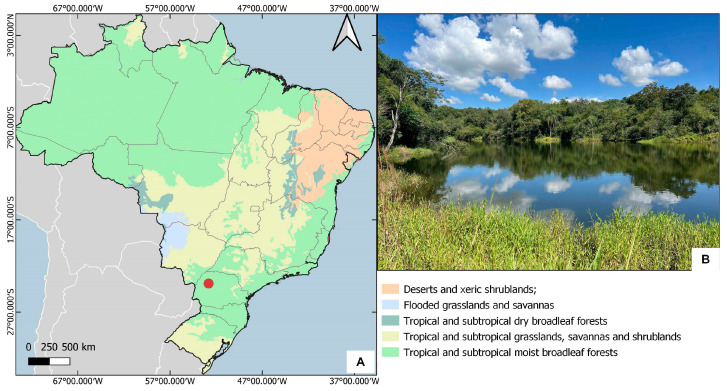
Distribution and habitat of *Hydrometra perobas* Cordeiro, Floriano & Moreira, sp. nov. (**A**) Location of the type locality (red circle). (**B**) Lagoon where we collected the holotype.

Type material. Holotype. BRAZIL—Paraná, Tuneiras do Oeste, zona de amortecimento da Reserva Biológica das Perobas, lagoa às margens da BR-487 (Figure 2); −23.8897, −52.8145; IV.2024; C.F.B. Floriano leg.; ♂, CEIOC 83747.

Micropterous male. BL 11.80; HL 3.40; ANTL 2.10; POSTL 1.00; ANTWmax 0.45; ANTWmin 0.22; POSTWmax 0.36; POSTWmin 0.30; CLL 0.15; CLWmax 0.12; HW 0.50; IOW 0.16; OL 0.25; EYE 0.16; ANT: I 0.42, II 1.10, III 2.75*, IV 1.65; LBL: I–II covered, III 2.27, IV 0.25; PL 1.85; PW 0.52; WiL 2.95; WiW 0.25; DIST1 1.00; DIST2 1.67; FORELEG, FEM 4.10; TIB 3.00; TAR: I 0.07, TAR II 0.30, TAR III 0.20; MIDLEG, FEM 3.16; TIB 3.20; TAR: I 0.07, II 0.37, III 0.25; HINDLEG, FEM 4.00; TIB 4.06; TAR: I 0.06, II 0.35, III 0.22; ABL 4.25; ABWmax 0.45; ABWmin 0.42; TERL: I–III covered, IV 0.80, V 0.75, VI 0.70, VII 0.60; TERW: I–III covered, IV 0.20, V 0.20, VI 0.20, VII 0.22; SVIII 0.75; PP 0.10 [* antennomere curved; measurement taken in a straight line].

### 3.1. Description

General color light brown to yellowish (Figure 1). Lateral margins of pronotum and acetabula slightly darker (Figure 1A); abdominal sterna III–VIII with distinctly darker areas (Figure 1D,E). Antennomere IV, distal margin of femora and tibiae, and tarsi dark brown (Figure 1F).

Head elongated; apex of anteocular region swollen (Figure 1A,B); anteocular region 2.1 times longer than postocular region; posterior pair of trichobothria reaching anterior margin of pronotum. Clypeus conate; lateral margins distally convergent; anterior margin rounded (Figure 1B). Eye well-developed, set on about posterior third of head (Figure 1A,D,F). Antenniferous tubercle with oblique distal margin. Antennomere I shortest, densely covered by short setae; III longest; IV 1.5 longer than II. Labium reaching postocular region of head. Venter of head not swollen around eyes.

Prothorax with a row of punctations adjacent to anterior margin; anterior lobe of pronotum without other punctations; posterior lobe with median row of punctations and many other punctations on each side more or less arranged in rows (Figure 1A); two irregular rows of punctations adjacent to posterior margin of propleuron. Proacetabulum with 18 circular punctations; mesoacetabulum with 17; metacetabulum with 10 (Figure 1G). Pro- and mesoacetabula each also with a large pit anteriorly and another one posteriorly located (Figure 1F,G). Forewing reduced, reaching abdominal mediotergite III (Figure 1A). Legs long, slender. Venter of thorax without carinae, tubercles or grooves.

Abdomen straight in lateral view (Figure 1F). Abdominal mediotergites III–VI about 4.5 times longer than wide, with glabrous shiny aspect. Abdominal mediotergite VII mostly opaque, without setae; with a small, glabrous, shiny circular area anteriorly; lateral margins diverging posteriorly (Figure 1C). Abdominal laterotergites slightly elevated. Abdominal sternum VI without distinct modifications (Figure 1E,H). Abdominal sternum VII with a pair of small elevations bearing dark setae (Figure 1E,H). Abdominal segment VIII cylindrical, without lateral tumescence, slightly constricted ventrolateraly (Figure 1E); posterodorsal process long, straight, with approximately 15% of total length of segment (Figure 1H).

Etymology. Named after the protected area Reserva Biológica das Perobas. The specific epithet is treated as a noun in apposition.

**Figure 3:**
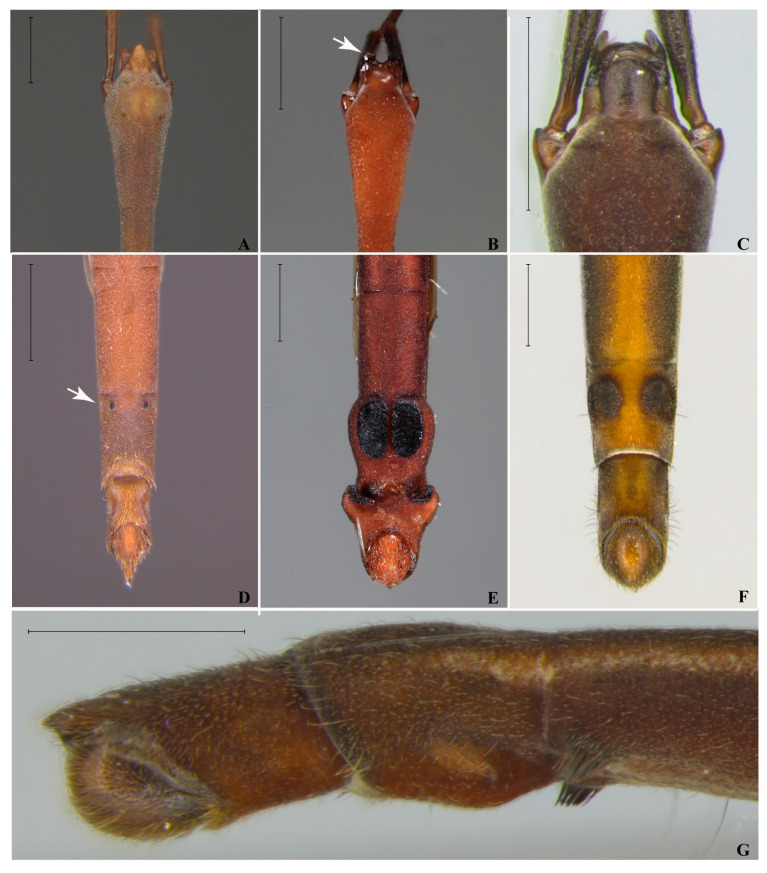
*Hydrometra*, key characters. (**A**–**C**) Anterior portion of head, dorsal view. (**A**) *Hydrometra argentina*. (**B**) *Hydrometra comata*, white arrow shows excavated anterior margin of clypeus. (**C**) *Hydrometra ruschii*. (**D**–**F**) Apex of abdomen, male, ventral view. (**D**) *Hydrometra argentina*. (**E**) *Hydrometra sztolcmani*. (**F**) *Hydrometra tuberculata*. (**G**) *Hydrometra ruschii*, apex of abdomen, male, lateral view. Scale bars: 0.50 mm.

Diagnosis. This new species can be distinguished from all congeners recorded from Brazil by the combination of the following features: (1) clypeus conate, with lateral margins distally convergent and anterior margin rounded (Figure 1B); (2) acetabula with more than ten circular punctations each (Figure 1G); (3) male abdominal sternum VI unmodified; and (4) male abdominal sternum VII with a pair of small elevations bearing dark setae (Figure 1E,H). Only two species that occur in the neighboring Argentina and Paraguay have not been recorded from Brazil, i.e., *H. quadrispina* Perez Goodwyn, 2001and *H. placita* Drake,1953 respectively. The former can be immediately recognized based on the male abdominal sternum VIII with four spines [8], whereas the latter has no punctations on the acetabula [9].

### 3.2. Key to the Hydrometra Recorded from Brazil

(modified from [10,11])

**Figure 4:**
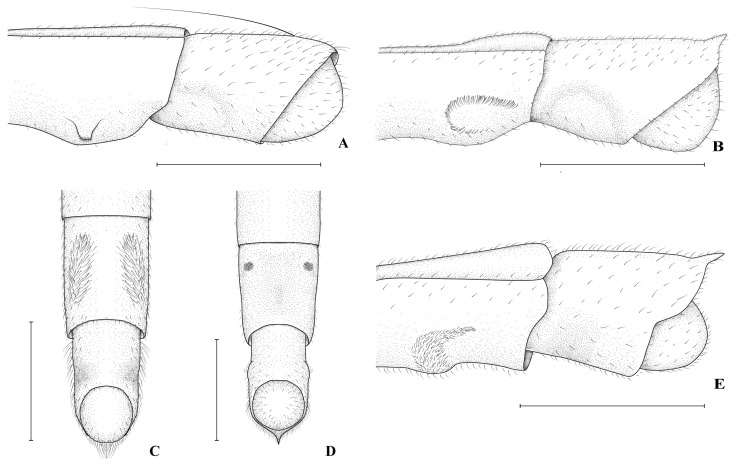
*Hydrometra*, apex of abdomen male. (**A**,**B**,**E**) Lateral view. (**C**,**D**) Ventral view. (**A**) *Hydrometra fruhstorferi*. (**B**) *Hydrometra guianana*. (**C**) *Hydrometra metator*. (**D**) *Hydrometra olallai*. (**E**) *Hydrometra caraiba*. Scale bars: 0.50 mm.

**Figure 5:**
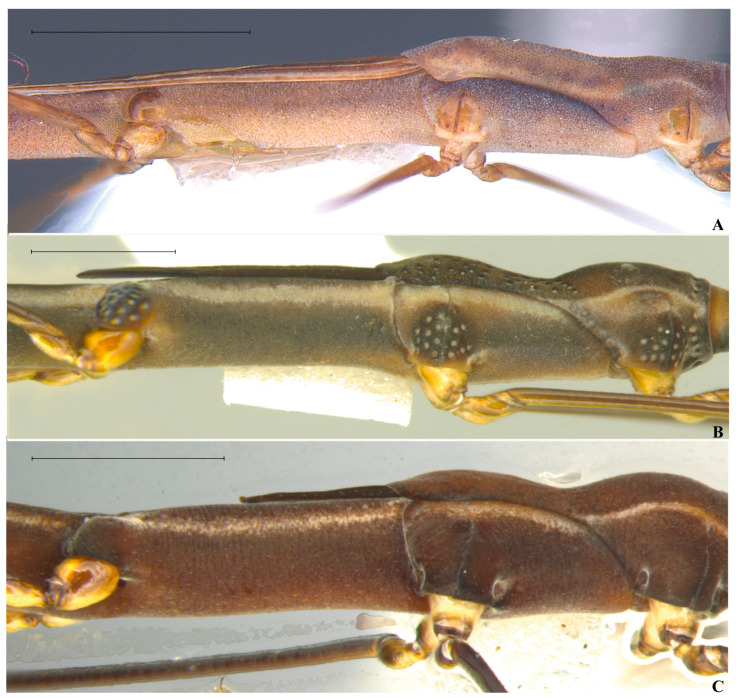
*Hydrometra*, thorax, lateral view. (**A**) *Hydrometra argentina*. (**B**) *Hydrometra tuberculata*. (**C**) *Hydrometra ruschii*. Scale bars: 1.00 mm.

1. Anterior margin of clypeus excavated (Figure 3B) …… *H. comata* Torre-Bueno, 1926
-. Anterior margin of clypeus acute, truncated or rounded, never excavated (as in Figure 3A,C) ……………………………………………………………………… 2
2. Male abdominal sterna VI and VII without projections or tufts of setae …………………………………………………..… *H. sapiranga* Moreira & Barbosa, 2013
-. Male abdominal sterna VI or VII with projections or tufts of setae (as in Figure 3D–G and Figure 4) ……………………………………………………………………………….... 3
3. Male abdominal sternum VI with a pair of tufts of setae near posterior margin (Figure 3G); male abdominal sternum VII without projections (Figure 3G) ………………………………………… *H. ruschii* Cordeiro, Rodrigues & Moreira, 2023
-. Male abdominal sternum VI unmodified; male abdominal sternum VII with tufts of setae or projections (as in Figure 3D–F and Figure 4) ………..…………………………… 4
4. Male abdominal segment VIII with large lateral hooks (Figure 3E) ……………………………………………………………... *H. sztolcmani* Jaczewski, 1928
-. Male abdominal segment VIII without large hooks (as in Figure 3D,F) …… 5
5. Pro- and mesoacetabula with 0–2 circular punctures or punctations (as in Figure 5A,C) ………………………………………………………………..…………….… 6
-. Pro- and mesoacetabula with at least 10 circular punctations (as in Figure 1G and Figure 5B) …………………………………………………………………………………………. 7

6. Male abdominal sternum VII with a large oval area bearing setae, where length of area is larger than half of sternum length (Figure 4C) ……………...… *H. metator* White, 1879
-. Male abdominal sternum VII with a pair of short, spinose projections anteriorly (Figure 3D) ……………………………………………………..… *H. argentina* Berg, 1879
7. Male abdominal sternum VII with a pair of mammilose projections (Figure 4A) or a pair of small elevations bearing dark setae (Figure 1E,H and Figure 4D) ………….… 8
-. Modifications of male abdominal sternum VII consisting of a pair of wider areas bearing distinct setae (Figure 3F and Figure 4B,C,E) ………………...………………… 10
8. Anterior half of male abdominal sternum VII with a pair of small elevations bearing dark setae (Figure 4D); posterior half with a central depression ………………………………………………………………….. *H. olallai* Mychajliw, 1961
-. Modifications of male abdominal sternum VII approximately at middle of segment (Figure 1E,H and Figure 4A); posterior half of segment without central depression ….. 9
9. Male abdominal sternum VII with a pair of mammilose projections joined by a wide transverse ridge (Figure 4A) ………… *H. fruhstorferi* Hungerford & Evans, 1934
-. Male abdominal sternum VII with a pair of small elevations bearing dark setae (Figure 1E,H) ……………… *H. perobas* Cordeiro, Floriano & Moreira, sp. nov.
10. Male abdominal sternum VII with a pair of large circular areas bordered by black setae (Figure 3F) ………………… *H. tuberculata* Cordeiro, Rodrigues & Moreira, 2023
-. Male abdominal sternum VII with a pair of clusters of setae with the shape similar to a semi-circle or a comma (Figure 4B,E) ……………………….………….… 11
11. Male abdominal sternum VII with a pair of clusters of setae on posterior half, with each cluster forming an almost closed circle (Figure 4B) …………………………………………………. *H. guianana* Hungerford & Evans, 1934
-. Male abdominal sternum VII with the pair of clusters of setae extending posteriorly, each cluster similar to a comma (Figure 4E) … *H. caraiba* Guérin-Méneville, 1857

## 4. Discussion

**Figure 6:**
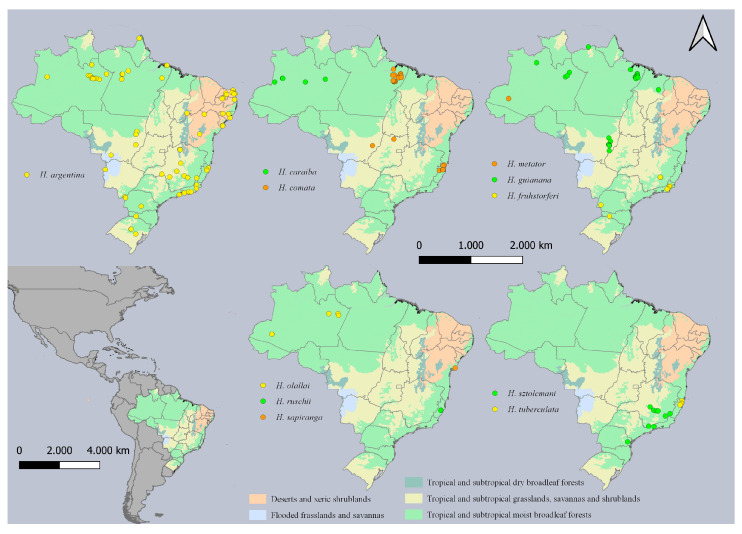
Geographic distribution of *Hydrometra* species recorded from Brazil. Distribution of *H. perobas* Cordeiro, Floriano & Moreira, sp. nov. depicted separately in Figure 2.

Our results increase the number of *Hydrometra* species recorded from Brazil (Figure 6) from 11 [12,13] to 12 (Figure 6). Furthermore, we record the first semiaquatic bug from the vicinity of Reserva Biológica das Perobas and elevate the number of species known from southern Brazil to four (*H. argentina*, *H. fruhstorferi*, *H. sztolcmani* and *H. perobas* Cordeiro, Floriano & Moreira, sp. nov.). Among them, *Hydrometra argentina* is distributed from Panama to Argentina and Uruguay, and one of the most common species of the genus in South America. Hydrometra *fruhstorferi* and *H. sztolcmani*, in turn, have been recorded from southeastern Brazil to northern and central Argentina, respectively [13].

Our revised key will aid those interested in identifying specimens of the genus collected in Brazil and hopefully prevent errors from being published in the future.

## 5. Conclusions

Although this study contributes to decreasing our knowledge gaps on the Brazilian fauna of semiaquatic bugs, much work remains. Many areas of the country remain completely unexplored and new species are constantly being discovered (e.g., [7,11]). Investing in field work performed by specialist taxonomists and faunal inventories is the most efficient way to overcome this.

## Data Availability

The data presented in this study are available in the article.
